# Unraveling the Peroxidase Activity in Peroxiredoxins: A Comprehensive Review of Mechanisms, Functions, and Biological Significance

**DOI:** 10.7759/cureus.66117

**Published:** 2024-08-04

**Authors:** Sana Qausain, Mohd Basheeruddin

**Affiliations:** 1 Biomedical Sciences, Allied Health Sciences, Datta Meghe Institute of Higher Education and Research, Wardha, IND; 2 Biochemistry, Jawaharlal Nehru Medical College, Datta Meghe Institute of Higher Education and Research, Wardha, IND

**Keywords:** pathways, oxidative stress, redox balance, peroxidase, peroxiredoxins

## Abstract

Peroxiredoxins (Prxs) are members of the antioxidant enzymes necessary for every living object in the three domains of life and play critical roles in controlling peroxide levels in cells. This comprehensive literature review aims to elucidate the peroxidase activity of Prxs, examining their roles and significance for organisms across various taxa. Ironically, the primary role of the Prxs is the peroxidase activity, which comprises the reduction of hydrogen peroxide and other organic hydroperoxides and decreases the risk of oxidative damage in the cells. The above enzymatic activity occurs through the reversible oxidation-reduction catalyzed by cysteine residues in the active site by forming sulfenic acid and reduction by intracellular reductants.

Structurally and functionally, Prxs function as dimers or decamers and show different catalytic patterns according to their subfamilies or cellular compartments. Compared to the mechanisms of the other two subgroups of Prxs, including 2-Cys Prxs and atypical Prxs, the 1-Cys Prxs have monomer-dimer switch folding coupled with catalytic activity. In addition to their peroxidase activity, which is widely known, Prxs are becoming acknowledged to be involved in other signaling processes, including redox signaling and apoptosis. This aversion to oxidative stress and regulation by the cellular redox state places them at the heart of adaptive cellular responses to changes in the environment or manifestations of diseases.

In conclusion, based on the data obtained and on furthering the knowledge of Prxs' structure and function, these enzymes may be classified as a diverse yet essential family of proteins that can effectively protect cells from the adverse effects of oxidative stress due to peroxidase activity. This indicates secondary interactions, summarized as peroxide detoxification or regulatory signaling, and identifies their applicability in multiple biological pathways. Such knowledge is valuable for enhancing the general comprehension of essential cellular functions and disclosing further therapeutic approaches to the diseases caused by the increased production of reactive oxygen species.

## Introduction and background

Peroxiredoxins (Prxs) evolved as a relationship among antioxidant enzymes in all life domains. Initially identified at the end of the 1990s as "thiol-specific antioxidant proteins," peroxiredoxins are now widely known as multifunctional proteins that exert profound control over many cellular processes [1].

Overview of peroxiredoxins

Peroxiredoxin is defined by its ability to scavenge hydrogen peroxide (H2O2) and other organic peroxides to neutralize the effects of peroxides in cells [2]. They fulfill this function using the conserved cysteine residues at their active sites, through which they are involved in redox cycles that are vital for their antioxidant capacity.

**Figure 1:**
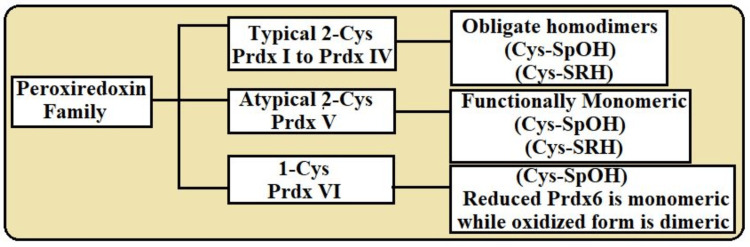
Peroxiredoxin family: subclasses of PRDXs This image was created by the author. Cys, cysteine; SpOH, commonly stands for serine-phosphoethanolamine; SRH, serine racemase homologue; Prdx6, peroxiredoxin 6.

The family of peroxiredoxins encompasses six distinct classes based on the number and arrangement of conserved cysteine residues involved in their catalytic mechanisms [3]. These classes include typical 2-Cys peroxiredoxins (Prdx I to Prdx IV), atypical 2-Cys peroxiredoxins (Prdx V), and 1-Cys peroxiredoxins (Prdx VI), as depicted in Figure 1, each exhibiting unique structural and functional adaptations suited to their specific roles in cellular physiology and response to oxidative stress [4].

Purpose of the review

As a result, this analysis provides a comprehensive overview of the peroxidase activity exhibited by each of the six peroxiredoxins in Table 1. The review's goals are to compile the most recent data and research findings. As a result, the following formulation of the research questions is possible.

**Table 1:** The following table systematically tabulates peroxidase activity in peroxiredoxin and focuses on its functions and importance in biological organisms This table was created by the author. Prx: peroxiredoxin; Nrf2: nuclear factor erythroid 2-related factor 2

Aspects	Details
Peroxiredoxin Types	1. Typical 2-Cys Prx
	2. Atypical 2-Cys Prx; 3. 1-Cys Prx
	2. Atypical 2-Cys Prx; 3. 1-Cys Prx
Peroxidase Mechanism	1. Typical 2-Cys Prx-of sulfenic acid (Prx-SOH) disulfide bond, Prx-S-S-Prx: red by thioredoxin
	2. Atypical 2-Cys Prx- Similar to the typical but the formation of intramolecular disulfide
	3. 1-Cys Prx– Generation of sulfenic acid intermediate (Prx-SOH) – Reduction by non-THiol reducers
Functions	1. Antioxidant Defense - Reduces H₂O₂, organic peroxides
	2. Cell Signaling- Regulates H₂O₂ signaling
	3. Redox Homeostasis - Maintains cellular redox balance
	4. Protein Protection - Protects proteins from oxidative damage
Biological Significance	1. Stress Response - increased for decoding oxidative stress
	2. Cell growth and differentiation – The gene controls information concerning the cell cycle and development
	3. Apoptosis Regulation - controls apoptosis signals
	4. Disease Association – Cancer, Cardiovascular diseases, Neurodegenerative disorders
Key Regulatory Mechanisms	1. Post-Translational Modifications - Phosphorylation, acetylation affecting activity
	2. Protein-Protein Interactions - Interactions with thioredoxin, other Prxs
	3. Gene Expression - Regulation by oxidative stress-responsive elements (e.g., Nrf2)
Research Applications	1. Biomarker Development- The levels of Prx as a marker of oxidative stress
	2. Therapeutic Targets – Therapy of disease through influencing Prxs
	3. Molecular Structural – X-ray Crystallography and Nuclear Magnetic Resonance to reveal the way things work
	4. Genetic Studies- Knockout and overexpression models to study function for genetic studies

Structural Insights

The analysis of various classes of peroxiredoxins explores the structural features and active site configurations regarding peroxidase activity.

Catalytic Mechanisms

Analysis of the strategies used by each group of peroxiredoxins, including particulars of the peroxide reduction process and the use of the invariant cysteine.

Biological Functions

Exploring the other functions of bovine serum albumin (BSA) and another antioxidant bimolecular that supplement the antioxidant protection functionality of organisms, including their functionality in all forms of redox signaling mechanisms, differentiation, apoptosis, and other forms of stress indication.

Biological Significance

Discussions on peroxiredoxins and their function in cellular redox control, along with an extension of the results of their dysregulation in diseases concomitant with oxidative stress.

In turn, addressing all these aspects within the review framework will help emphasize the significance of peroxiredoxins as multipurpose enzymes that maintain cellular homeostasis and protect the organism from oxidative stress. This integration of knowledge may also help inform further research and areas for treatment involving the use of peroxiredoxins in diseases associated with oxidative stress.

## Review

Structural and catalytic mechanisms of peroxiredoxins

Peroxiredoxins (Prxs) represent one of the most important antioxidant enzymes in cells and protect them from oxidative stress [5]. This section describes their general characteristics, the catalytic cycle, and differences between isoforms that help understand the functional variety and the mechanisms that execute their tasks.

Common Structural Features

The enzyme's active site contains peroxidative cysteine (Cys-P), which is crucial for initiating the catalytic cycle when hydrogen peroxide (H2O2) or organic hydroperoxides are used. Cys-P is oxidized during this step to produce Cys-SOH, a sulfenic acid intermediate essential for later phases of the catalytic cycle [6]. After peroxide reduction, resolving cysteine (Cys-R), which is exclusively found in conventional 2-Cys peroxiredoxins and some atypical 2-Cys peroxiredoxins, forms a disulfide bond with Cys-P to reduce the sulfenic acid intermediate. To restore the active form of peroxiredoxin (Prx), this disulfide bond is subsequently reduced by thioredoxin or other cellular reducing agents [4].

The preserved active site architecture of prxs is typified by a structural fold comprising β-sheets and α-helices that form a reactive center loop (RCL) that houses Cys-P and Cys-R. This conserved design is crucial for interactions with regulatory proteins, catalytic efficiency, and substrate recognition [7].

Catalytic cycle

Peroxiredoxins (Prxs) catalyze a cycle of consecutive processes that detoxify peroxides. These procedures guarantee the effective elimination and reduction of hazardous peroxide molecules.

Reduction of Peroxide

Peroxidation is the initial stage of the catalytic cycle of peroxiredoxins. Peroxidatic cysteine (Cys-P) combines with hydrogen peroxide (H2O2) to generate a sulfenic acid intermediate (Cys-SOH). In the second stage, resolution, in typical 2-Cys peroxiredoxins, the sulfenic acid intermediate forms an intermolecular disulfide bond (Cys-S-S-Cys) with the resolving cysteine (Cys-R) of a neighboring subunit. Lastly, thioredoxin or other cellular reducing agents reduce this disulfide bond in the reduction step, renewing the active site cysteine residues (Cys-P and Cys-R) for a subsequent catalytic cycle [8]. 

The Power of PRDXs as Catalysts

According to the existing literature, the Cys-SPH attacks the peroxide substrate and is oxidized to a Cys-SOH during the initial stage of the catalytic cycle. All three PRDX classes seem to share this step [9,10]. Although the precise catalysts driving this reaction are still unknown, an acid and a base are likely needed for the peroxide breakdown to protonate the remaining group, RO-, and deprotonate the peroxidative cysteine. All PRDXs have an active-site Arg that slightly reduces the cysteine's pKa to preserve the peroxidative cysteine's thiolate state [11].

**Figure 2:**
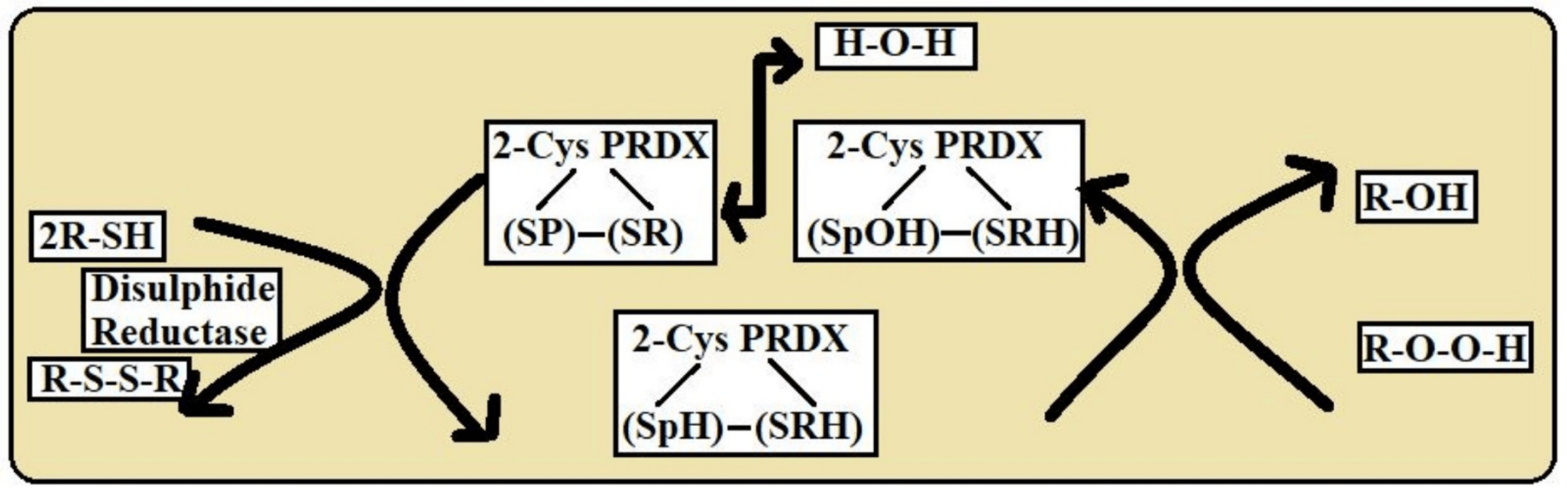
The above diagrams show the catalytic mechanism of typical 2-Cys PRDX This image is the author's own creation. 2-Cys PRDX-2: Cysteine Peroxiredoxin; SPOH: Sulfiredoxin Peroxidatic Cysteine; SRH: Sulfonic Acid; SP: Peroxidatic Cysteine; SR: Resolving Cysteine

The three PRDX classes are separated in the secondary stage of the peroxidase reaction. The two redox-active cysteines in the most prevalent kind of PRDXs, known as conventional 2-Cys PRDX, are the peroxidative cysteine (usually found at residue 50) and the resolving cysteine (commonly found close to residue 170). The two homodimers comprising typical 2-Cys PRDX, as depicted in Figure 2, have the same active sites [12]. Cys-SRH, often found at the C-terminus of one subunit, interacts with Cys-SPOH, positioned on another subunit, during the second stage of the peroxidase reaction, and 4. Following this condensation phase, one of the numerous cell-specific disulfide reduction processes reduces the stable intersubunit disulfide bond.

Regulation and Efficiency

Prxs are regulated by the cellular redox environment, where oxidative stress conditions promote their oxidation and activation while reducing conditions favor their reduction and inactivation. Oligomeric states (e.g., dimers, decamers) and interactions with regulatory proteins further modulate their catalytic efficiency and biological functions [13].

Isoform-Specific Variations

**Figure 3:**
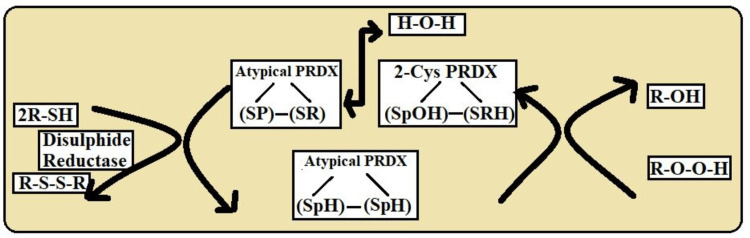
The above diagram show the catalytic mechanism of atypical 2-Cys PRDX This image is the author's own creation. 2-Cys PRDX-2: Cysteine Peroxiredoxin; SPOH: Sulfiredoxin Peroxidatic Cysteine; SRH: Sulfonic Acid; SP: Peroxidatic Cysteine; SR: Resolving Cysteine

Peroxidatic cysteine (Cys-P) and resolving cysteine (Cys-R) are the catalytic sites of typical 2-Cys peroxiredoxins (Prxs), which form homodimers. These enzymes regulate redox processes and antioxidant protection; they are often present in eukaryotes [4]. To address particular cellular systems or stressors, atypical 2-Cys Prxs are depicted in Figure 3, displaying changes in their design or mode of functioning [14]. 

**Figure 4:**
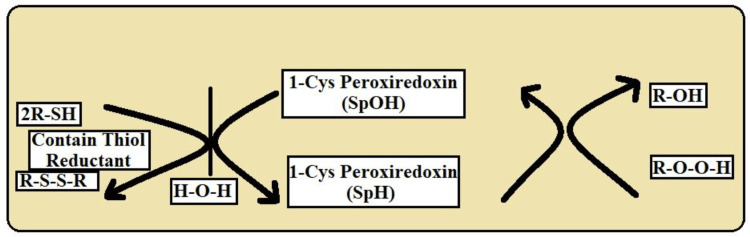
The above diagram shows the catalytic mechanism of 1-Cys PRDX This image is the author's own creation. 2-Cys PRDX-2: Cysteine Peroxiredoxin; SPOH: Sulfiredoxin Peroxidatic Cysteine; SRH: Sulfonic Acid; SP: Peroxidatic Cysteine; SR: Resolving Cysteine

By comparison, 1-Cys Prxs, as seen in Figure 4, which frequently lack a resolving cysteine and hence form monomers, go through a brief and straightforward redox cycle in which cellular thiols immediately reduce Cys-SOH. Prokaryotes and certain lower eukaryotes have a higher prevalence of this type [15].

Knowledge of these structural and catalytic entities gives a broad perception of how Prxs positively influence the processes of redox signaling, oxidative stress, and physiological functions. Due to isoform-specific differences, their sensitivity towards various biological environments is evident, and thus, for treating diseases associated with oxidative stress, these molecules present targets for therapy.

Regulation of peroxidase activity in peroxiredoxins

It is well understood that peroxiredoxins (Prxs) are involved with the limited antioxidant defense of the cell through their peroxidase activity in the elimination of peroxides. This section focuses on the various ways Prx activity can be controlled: through PTMs, oligomerization, redox state, and feedback regulations [16].

Post-Translational Modifications (PTMs)

Peroxiredoxins (Prxs) are phosphorylated at particular locations, impacting their enzymatic activity and functionality. For example, phosphorylation might change the oligomerization partners or the way redox signaling proteins are compartmentalized, which can affect the redox processes in the cell as a whole [17]. Acetylation alters stability, enzymatic effectiveness, and responsiveness to oxidative stress, affecting Prx activity at the protein, subunit, or residue level. Prxs' ability to form oligomeric complexes and interact with other proteins may be impacted by this alteration, which may affect how well Prxs functions in the fight against oxidative stress [13].

Prx activity is additionally regulated by additional post-translational modifications (PTMs) like glycosylation, ubiquitination, and S-nitrosylation. Prx shape, stability, and turnover rate can all be changed by these changes, which affect how well they bind peroxides and shield cells from oxidative stress [18].

Oligomerization and Redox State

Oligomeric state: The Prxs can work in different oligomeric states, such as dimers and decamers, affecting their catalytic properties and functions. Thus, the transition between the oligomeric states may be modulated by changes in pH, temperature, and/or binding to specific ligands or chaperones [15].

**Figure 5:**
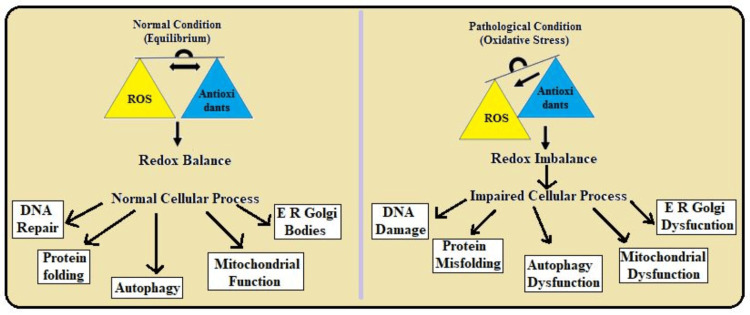
Description of normal and dysfunctional cellular processes contributed by redox imbalance This image is the author's own creation. ROS: Reaction oxygen species; ER: Endoplasmic reticulum

Redox regulation: The process of oxidoreduction in Prxs is characterized by the reversible oxidation of cysteinyl residues. Normal cellular processes are illustrated in Figure 5; redox homeostasis is upheld by the generation of ROS and antioxidant activity [19]. These balances enable ROS to act as a signaling agent capable of modulating numerous cellular processes such as cell division and differentiation, cell division, and apoptosis without toxicity. Mitochondria produce ATP while at the same time minimizing the production of ROS, and the existing ROS are scavenged by antioxidants such as SOD and catalase. This guarantees that metabolic processes, energy production, and other cellular repair processes are fixed and take place effectively to ensure that healthy and whole cellular functioning is achieved [20]. 

Figure 5, on the other hand, illustrates impaired cellular processes due to redox imbalance. This is characterized by ROS production being greater than the body’s antioxidant mechanisms, thus causing oxidative stress. This results in alterations in cellular signaling and dysfunction of mitochondria by generating an oxidative change in DNA, proteins, and lipids [21]. Consequently, cellular energy production may be compromised; higher levels of mutations and, in some cases, misfolded proteins will be observed. It is also considered that chronic oxidative stress can lead to inflammation that results in diseases such as cardiovascular diseases, neurodegenerative diseases, diabetes, and other diseases. In summary, redox deregulation has appalling effects on cells' internal environment and strikes the refined mechanics of cell vitalities. It imposes deterioration of normal function and disease advancement [22].

Feedback Mechanisms

Some peroxiredoxins (Prxs) can self-regulate their activity levels by using feedback mechanisms based on their redox state. This is achieved by generating reduced power by their peroxidase activity, which results in reduced thioredoxin (Trx), which can either influence Prx activity or take part in other redox signaling pathways [23]. Peroxiredoxins (Prxs) are integrated into cellular redox networks, interacting with other redox-sensitive proteins and signaling molecules to enable sophisticated physiological responses to oxidative stress and different situations. They are also regulated by and contribute to the regulation of the system [19].

Peroxiredoxins (Prxs) are involved in the redox control of Prx activities that preserve cellular redox balance because they can directly interact with regulatory protein factors like chaperones and kinases. These factors can alter the stability and intensity of Prxs, depending on the degree of cellular stress [24]. Knowledge of these regulatory mechanisms explains how Prxs respond to the changing needs of the cell as they combat oxidative stress. Abnormality in the activity of Prx caused by changes in PTMs, oligomers, or redox status is involved in various diseases where oxidative damage plays a role, with a proposal that Prxs may be suitable targets for drugs used in the treatment of oxidative stress-related diseases.

Functional roles of peroxiredoxins in cellular physiology

Initially, Prxs had been considered exclusive antioxidant enzymes; however, they now participate in various consequential pathways in cellular physiology. This section focuses on their protective functions against oxidative stress, signaling and regulating cell division, and cell death.

Antioxidant Defense

Peroxiredoxins (Prxs) protect cells from oxidative stressors caused by reactive oxygen species (ROS) by decreasing hydrogen peroxide and organic hydroperoxides [25]. Particularly in situations of oxidative stress brought on by external pollutants or metabolic changes, Prxs sustain cellular function and survival by preserving redox equilibrium and minimizing oxidative damage to biomolecules such as proteins, lipids, and DNA [26].

Redox Signaling

Using reversible thiol-oxidation and -reduction events, peroxiredoxins (Prxs) control the redox balance of cells. This regulates gene expression, enzyme activity, and cellular signaling, among other downstream redox signaling processes. Prxs function as redox regulators, affecting redox-sensitive proteins and transcription factors linked to immune responses, cellular differentiation, and stress adaptation. Additionally, by integrating into redox networks, Prxs aid in coordinating the cell's response to oxidative stress and other oxidative signals [19].

Cell Cycle and Apoptosis

Cell cycle and apoptosis regulation: Prxs are involved in cell cycle regulation by maintaining cellular ROS homeostasis. Thus, the appropriate redox balance, which Prxs regulate, is significant for the cell cycle checkpoints and DNA damage response. In this context, Prxs control apoptosis, functioning both as a promoter and an inhibitor. Some can decrease the likelihood of apoptosis by adjusting the redox balance to fend off oxidative stress. On the other hand, under some circumstances, Prxs act as anti-apoptotic proteins through several redox signaling cascades [27].

Implications in disease: Dysregulation of Prx activity or expression implicates it within several diseases, such as cancer, neurodegenerative diseases, cardiovascular diseases, and metabolic diseases. Regarding Prx functions in cellular physiology, it can be assumed that information about this protein helps understand diseases and the potential treatment based on oxidative stress-interconnected pathways [18].

Thus, peroxiredoxins are to be seen as the universally essential enzymes for cellular functioning that are directly linked with antioxidant defense, signaling using redox reactions, regulation of the cell cycle, and apoptosis. Instead, their functions go as far as modulating cellular signaling in physiology and pathology, which is regulated by oxidative stress.

Biological significance in health and disease

PRx is expressed in every mammalian tissue and has an active role in various physiological and pathophysiological processes bilaterally, meaning it has enormous potential to help diagnose and treat diseases.

Prxs in Normal Physiology

To maintain redox homeostasis, which is necessary for healthy cellular function in metabolic activities, signaling, and cell division, peroxiredoxins (Prxs) must remove reactive oxygen species and prevent oxidative stress [19]. Prxs function as primary antioxidants, defending against oxidative stress caused by environmental stimuli and endogenous metabolic activity. They also participate in redox signaling pathways that control immune responses, cellular differentiation, gene expression, and stress adaptation [28,29].

Prxs in Pathological Conditions

**Figure 6:**
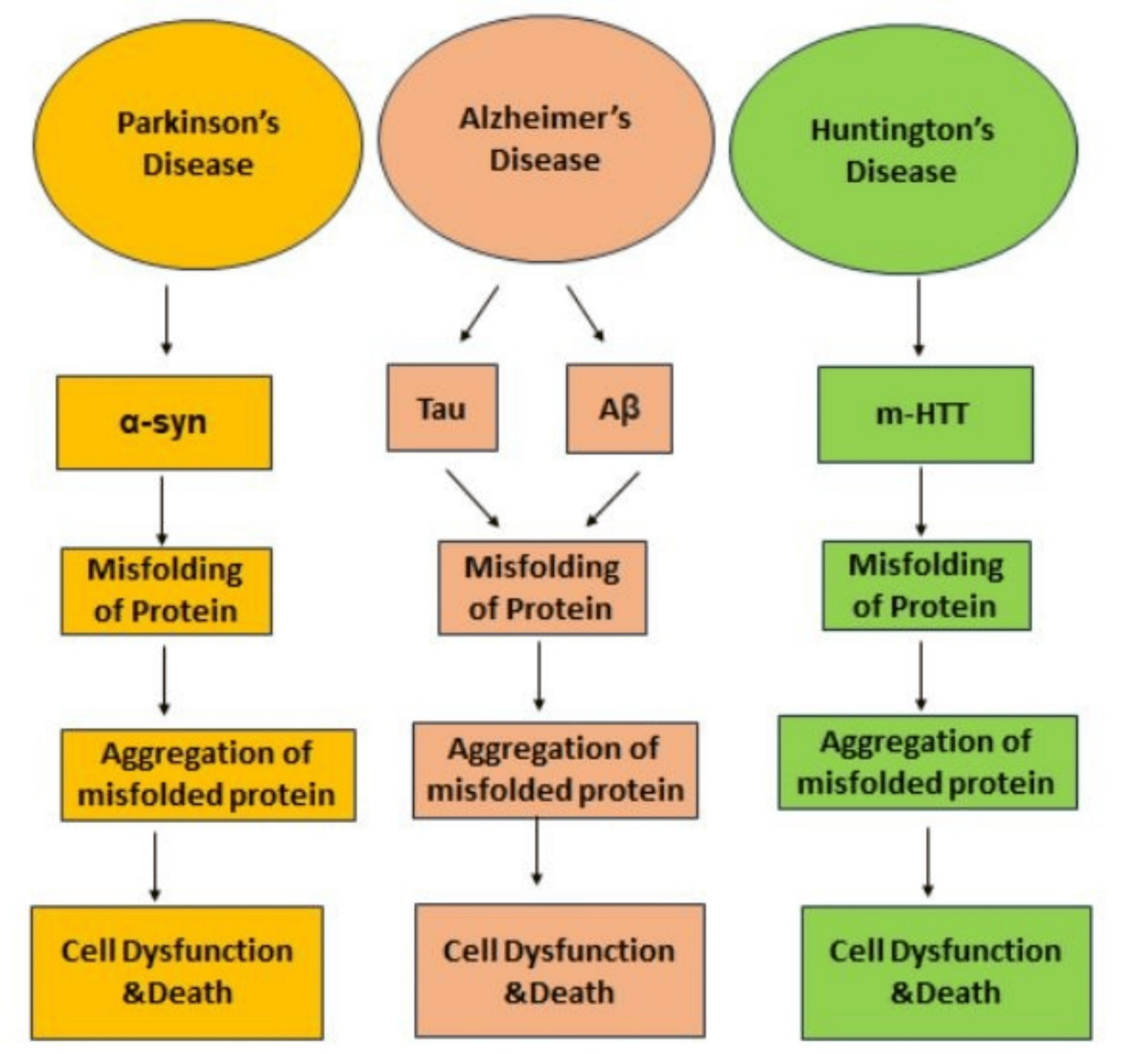
Common pathogenic features of neurodegenerative illnesses include cell malfunction, neuronal death, and the buildup of specific proteins that form insoluble aggregates inside and/or between neurons This image is the author's creation. α-syn: alpha-synuclein; Aβ: Amyloid-beta; mHTT: mutant huntingtin protein

Cancer progression is linked to the dysregulation of peroxiredoxins (Prxs), whereby alterations in redox signaling and elevated levels of reactive oxygen species (ROS) lead to genomic instability and resistance to apoptosis. Particular Prx isoforms have been associated with metastasis and cancer cell survival, indicating their function in establishing tumors [30]. Reduced Prx expression or activity is correlated with the severity of neurodegenerative illnesses, including Alzheimer's, Parkinson's, and Huntington's disease, as depicted in Figure 6 [31]. Prxs protect against oxidative damage and preserve neuronal function in these conditions.

Additionally, Prxs protect cardiovascular tissues from oxidative damage, and dysregulated Prx expression has been linked to diseases like hypertension, atherosclerosis, and myocardial infarction [32]. Prxs also control the generation of cytokines and redox-sensitive transcription factors, which modulate inflammatory responses. Inflammatory diseases such as rheumatoid arthritis and inflammatory bowel disorders are known to have changed Prx levels [33].

Prxs as biomarkers in various diseases

Peroxiredoxins are integral to normal cellular physiology through their roles in redox homeostasis and antioxidant defense. Deregulations of Prx function contribute to the pathogenesis of various diseases, making them potential biomarkers for disease diagnostic markers, prognosis markers, risk markers, predictive markers, inflammatory markers, and biomarkers for infection severity, as depicted in Table 2, aimed at restoring redox balance and mitigating oxidative stress-related damage [34].

**Table 2:** Peroxiredoxins (Prxs) as biomarkers in various diseases This table was created by the author.

Disease/Condition	Peroxiredoxin Isoform	Biomarker Role	Clinical Implication	Reference
Cancer	Prx 1	Prognostic Marker	Elevated levels linked to poor prognosis and aggressive tumor behavior	Nicolussi A, et al. [35]
Neurodegenerative Diseases	Prx 2	diagnostic marker	Increased expression associated with oxidative stress in neurodegeneration	Szeliga M. [36]
Cardiovascular Diseases	Prx 3	Risk marker	Correlation with mitochondrial dysfunction in heart disease	Jeong SJ, et al. [37]
Diabetes	Prx 4	Predictive marker	Elevated levels predict the onset of diabetes complications	Thomas PB, et al. [38]
Inflammatory Diseases	Prx 5	Inflammatory marker	Increased expression linked to chronic inflammation	Chen L, et al. [33]
Infectious Diseases	Prx 6	Biomarker for Infection Severity	Higher levels found in severe cases of infection	Landry A, et al. [39]

Therapeutic potential of targeting peroxiredoxins (Prxs)

Peroxiredoxins (Prxs) present promising therapeutic targets due to their pivotal roles in redox regulation and antioxidant defense mechanisms. This section explores strategies for modulating Prx activity, progress in drug development, and the challenges and opportunities in targeting Prxs for therapeutic purposes.

Targeting Prxs in Therapy

Developing small chemical inhibitors that specifically restrict the activity of peroxiredoxin (Prx) or interfere with their interaction with reducing partners, like thioredoxin, is a potential strategy. In cancer cells or other tissues with increased oxidative stress, these inhibitors may impair Prx's antioxidant defense, which could affect the course of the disease [40].

Conversely, boosting Prx expression or Prx enzyme activity may be advantageous for cellular antioxidant defenses. Since oxidative stress plays a role in the development of neurodegenerative diseases and cardiovascular disorders, this strategy may be especially useful in controlling such conditions [31]. Furthermore, given that Prxs play varied roles in various disease scenarios, focusing on their isoform-specific actions may provide more targeted therapeutic approaches, particularly in neuroprotection and cancer [41].

Drug Development

Research into peroxiredoxin (Prx)-targeted drugs has advanced considerably. The most studied areas include how these antioxidant enzymes can protect cells and regulate cellular signaling. Scholars have discovered Prx inhibitors and activators with likely therapeutic uses for cancer, neurodegenerative disorders, and cardiovascular illnesses [42].

In cancer, it is desired to raise oxidative stress in the tumor cells through Prx inhibitors and concomitantly raise the effectiveness of chemotherapy and radiotherapy. Neurodegenerative diseases like Alzheimer’s and Parkinson’s are associated with oxidative damage, and Prx activators assist in reducing the same. Due to rapid developments in structural biology and high-throughput screening, there is the possibility of discovering small molecules that selectively activate or inhibit Prx molecules. While many of these Prx-targeted compounds are still in their early stages of development, preclinical work has indicated that these drugs could be worthy of further work in clinical trials and later potential treatments [43].

Challenges and Opportunities

The following are some challenges when focusing on peroxiredoxins (Prxs) in drug development. Due to the presence of six isoforms of Prx in the human genome, their redundancy and compensation for losing an individual Prx or enhancing its activity hamper attaining selective therapeutic goals. The close relationship between Prxs and other thiol-based antioxidant enzymes is one of the factors that has contributed to the challenge of achieving selectivity for Prx-specific drugs. Furthermore, because of Prxs’ primary function in protecting live cells from oxidative stress, their blockade results in adverse effects such as toxicity in non-target cells, which presents a therapeutic challenge. Problems, such as the targeted delivery of Prx-acting drugs to the tissues or cellular compartments where Prxs operate, continue to be challenging [13].

However, there are great possibilities for treating diseases with the help of targeting Prxs. Therefore, further investigation of Prxs seems promising for designing new therapeutic strategies. In cancer treatment, anti-Prx can selectively increase ROS production, specifically in cancer cells, and thus facilitate cell death modalities such as chemotherapy or radiotherapy. In neurodegenerative diseases, regulation of Prx activity may help to avoid oxidative stress, which can decelerate the development of such diseases as Alzheimer’s disease and Parkinson’s disease [44]. Concerning cardiovascular health, therapies that target Prx may relieve oxidative stress diseases, hence opening new horizons for management.

Furthermore, it was suggestively claimed that Prx inhibitors might be used in conjunction with other agents, the combination of which would be more potent than each compound taken alone. Prx levels and activity might also be employed as molecular markers of disease progression and treatment efficacy, which would contribute to improving patients’ tailor-made treatment. Thus, while targeting Prxs is challenging, the potential therapeutic benefits make it a promising area for future drug development [45].

## Conclusions

Therefore, this review has aimed to expound on the complex peroxidase activity of Prxs as antioxidant proteins responsible for the maintenance of cellular redox homeostasis and protection against oxidative stress. With the help of the descriptions of the catalytic processes, we exposed the specific cycle with cysteine residues that make Prxs effective in peroxide reduction. This peroxidase activity is essential for metabolism, protection against oxidative stress, and regulating redox signaling processes within human cells. Due to the multifaceted role of Prxs in numerous cellular contexts, studying Prxs’ function in health and disease is of high relevance.

However, the involvement of Prxs does not end with such enzymes’ action; it is proven by the fact that they also have other roles that are biologically important. Prxs are involved in numerous cellular processes, including cell death, cell division, and immune reactions. For this reason, Prxs have sensing and regulatory capabilities with regard to hydrogen peroxide concentration. They become deregulated to play a role in various pathological states like cancer, neurodegeneration, and cardiovascular diseases. This knowledge of the many parts of Prxs is an apparent platform for the development of therapeutic strategies for mitigating the impact of oxidative stress conditions. This massive review highlights the need for more research work due to the variance in structure and functions of Prxs to gain its maximum potential in clinical uses.
